# A Commentary on Pokropski's Functionalist Reading of Husserlian Phenomenology

**DOI:** 10.3389/fpsyg.2021.614842

**Published:** 2021-03-23

**Authors:** Witold Płotka

**Affiliations:** Institute of Philosophy, Cardinal Stefan Wyszyński University in Warsaw, Warsaw, Poland

**Keywords:** functionalism, transcendentalism, eidetics, eidetic variation, reduction, noema

## Introduction

In his recent study, Pokropski argues that Dreyfus's famous (or infamous) cognitivist reading of Husserl's theory of mind is partly misleading, specifically, as a strong computationalism. The strong computationalist reading of Husserl, according to Pokropski (2020, p. 871), holds that (1) the theory of noema is a sort of adumbration of contemporary computationalism, such as Fodor's representational theory of mind (RTM); (2) this mean, however, that the noema functions as a mediatory entity (just as Fodor's mental representations); finally, (3) the noema (or mental representations) do not require a mind's physical realization. Pokropski follows McIntyre in arguing that the strong computationalist reading of Husserl's phenomenology is implausible. In this regard, Pokropski's arguments reflect the on-going polemic in the literature (e.g., Drummond, 1990, 2012; Zahavi, 2004, 2017; Szanto, 2012; Płotka, 2017). Yet, Pokropski's original idea and his key insight here is to develop an alternative functionalist reading of Husserl's phenomenology. He holds that Husserl applies an explanatory strategy of decomposition which resembles the method of functionalism. To show this, Pokropski analyzes Husserl's project of phenomenological psychology which seems to address these claims. In the end, he argues that the project allows a non-reductive naturalization of phenomenology.

Pokropski (2020, p. 883) claims that “functional phenomenology describes and analyzes processes which contribute to experiences of a certain type. Functional phenomenology can be located at the same level as phenomenological psychology, which is different from the transcendental level.” In my opinion, however, the thesis is questionable. Pokropski takes for granted that phenomenological psychology and transcendental phenomenology are *clearly* differentiated and the former can be developed regardless of the latter. In the following commentary, I will argue that Pokropski's claim is unjustified for methodological reasons. In this regard, I attempt to show that (1) Pokropski fails in defining the method of functional phenomenology and, what follows, he does not employ the method used by Husserl. Against this background, (2) I will suggest an alternative approach to Pokropski's reading.

## The Eidetic and The Transcendental in Phenomenology

Pokropski's view on Husserl's phenomenological psychology can be summarized as follows: (1) eidetic (or phenomenological) psychology is a discipline that mediates between transcendental phenomenology and experimental psychology; (2) Husserl's psychology applies the reduction—called “eidetic,” or “phenomenological”—that is *different* from the method of transcendental phenomenology; finally, (3) eidetic (or phenomenological) psychology describes the structure of experience as the noetic–noematic correlation.

The *first* thesis follows directly from Husserl (1977, p. 31–33, 144; 1997, p. 95–99), who holds that eidetic psychology and transcendental phenomenology have to be kept most rigorously distinct. For Pokropski the thesis is justified since, as he argues, both disciplines use distinct methods. Yet, the *second* thesis is problematic. Although Pokropski (2020, p. 874) explains that the *eidetic* reduction consists in “excluding” “all reference to the physical basis of the mental” and in focusing on what is “hidden” in the natural attitude, he does not provide a clear definition of *transcendental* reduction. The difference, however, is crucial for his argument. One might expect what does it mean to perform the eidetic reduction as distinct from the transcendental one. In this regard, I would suggest that the lack is not accidental and it goes back to Husserl himself. The doctrine of reduction in Husserl is, of course, complex (e.g., Lobo, 2013; Theodorou, 2015, p. 17–66; De Santis, 2020), but it is misleading to claim that he operates with clear-cut methods. In contrast to Pokropski, then, I hold that the eidetic–transcendental divide in Husserl is not sharp. Rather, eidetic and transcendental methods are closely related as elements of Husserl's general research strategy.

Of course, eidetic psychology investigates the *essences* of mental states, yet those states are given *not* as psychic tokens (or examples of natural phenomena), but rather as pure experiences. Otherwise, Husserl's eidetics would consist in gradual and inductive generalizations. Simply stated, eidetics would then become a natural science. Contrary to Pokropski, Husserl's eidetic approach is *transcendental* through and through. This means that eidetic approach requires the reduction. Yet, reduction in phenomenology does *not* exclude—as Pokropski suggests—anything, say the natural or the world. Rather, it leads back (*re-ducere*) to what is experienced as such, i.e., in its essence. Accordingly, it is a mere change in focus. Reduction thus understood makes possible a systematic analysis of the world–subjectivity correlation: whereas eidetic tools illuminate some transcendental concepts, this is possible *only* within the transcendental attitude which, in turn, gives leading clues for further eidetic inquiry. The object for eidetic psychology is transcendental, or purified consciousness. For Husserl (1997, p. 247), as he puts it in the *Amsterdam Lectures*, the reduction “opens up to him or her [i.e., the phenomenologist] the field of transcendental experience and the eidetics of the transcendental.” Thus, the eidetics explores the transcendental.

This leads to the *third* thesis. For Pokropski (2020, p. 874), non-transcendental psychologists comprehend lived experiences through the noesis–noema lenses. Yet, the thesis is contradict with the idea that they do *not* adopt the transcendental attitude. Contrary to Pokropski's suggestion, both the noesis and the noema are accessible, as Husserl (1983, p. 213) puts it, only in “the frame of the transcendental reduction.” Pokropski's position, then, is in fact anti-Husserlian. The theory discussed by Pokropski suggests that eidetic psychology can ignore transcendental reduction and transcendental attitude, but this is false (at least in Husserl). If he wants to include the noesis and the noema into the field of eidetic (or phenomenological) psychology, he has to accept that these elements are accessible only due to transcendental reduction.

These ideas in mind, it can be concluded that Pokropski fails to employ an appropriate method. For instance, he considers an example of the perception of an apple tree (Pokropski 2020, p. 880), yet the exemplary experience is not for him—as for Husserl—the basis for the further procedure of eidetic variation. For Husserl (1997, p. 93), “factuality serves only as an exemplar, a basis for the free variation of possibilities, whereas what we are seeking to ascertain is the *invariant* that emerges in the variation, the *necessary typical form*, which is bound up with the ability to be thought.” In his lectures on phenomenological psychology, Husserl shows that individuals and the world itself have their proper form which can be filled with particular content. These forms can be studied in pure fantasy where “factual experience gives me only an exemplary beginning for the style of free fantasies which I shape from it, without otherwise employing it as something to be accepted” (Husserl, 1977, p. 53). Hence, according to Husserl, pure fantasy allows for an *a priori* which is understood as “the invariable” in a free variation of experience. The use of examples by Pokropski does not fit Husserl's requirements. He describes the particular phenomenon of perceiving an apple tree, but the description concerns the specific mental state and it does not address the free variation of possibilities. Rather, Pokropski refers to some *results* of the procedure used by Husserl, but he does not employ the method described by Husserl.

## An Alternative Approach to Pokropski's Reading

Pokropski is skeptical of transcendental phenomenology. He seems to assume that the transcendental, i.e., non-natural approach makes naturalization of phenomenology difficult, if not impossible [for discussion, see Ramstead (2015)]. In this fashion, Pokropski develops a non-transcendental psychology which, however, has its object—the noesis–noema correlation—accessible only from a transcendental viewpoint. Next, the project examines intentional functions [called by Pokropski (2020, p. 876–877) “noetic functions” interchangeably, i.e., functions of consciousness which consist in constituting, or producing its object], as defined in Husserl's (transcendental) *Ideas I*, yet in order to decompose them one has to use theoretical tools of the mereological theory as formulated in the (non-transcendental) *Logical Investigations*. Indeed, the latter theory is unable to account for the theory of *Ideas I*, since it leads toward a new psychologism, as Husserl (1970, p. 354, fn. 24) warns in the second edition of his *Investigations*. Pokropski (2020, p. 883) tries to define functional phenomenology as a form of phenomenological psychology, which is different than transcendental phenomenology; yet, as shown, Pokropski's project still uses transcendental tools and as such it is held within the transcendental framework. Thus, it shall come as no surprise that Pokropski's project is inconsistent.

Given these difficulties, one can still argue that Pokropski's problems reflect Husserl's own attempts (and his followers) to define phenomenology as distinct from psychology [see e.g., Spiegelberg (1972), Fisette (2018), Płotka (2020)]. In the end, let me suggest an alternative approach which can contribute to the problem discussed by Pokropski. In the paper, he uses some exemplary descriptions of psychic phenomena provided by Husserl, e.g., time–constitution, or the world–body correlation, and claims that these descriptions contribute to cognitive science. I think that it would be more promising to *suspend* Pokropski (2020, p. 878) claim that functional psychology *precedes* the empirical one and that it has to adapt the tool of reduction. In this approach, discussed recently by Zahavi (2019), cognitive science can refer to and be inspired by some findings, or descriptions provided by phenomenologists, yet a cognitive scientists can ignore the epoché and the reduction in their own studies.

In general, Zahavi critically examines van Manen's and Giorgi's demand of an adaption the reduction to the human sciences, especially within qualitative research. In this regard, Zahavi refers to the tension present in Husserl's psychological writings which makes hard to address the question whether the epoché and the reduction are really essential to phenomenological psychology. According to Zahavi (2019), “there is something intrinsically self-undermining in the proposal that phenomenological psychology, understood as a distinct qualitative research method different from both naturalistic psychology and transcendental phenomenology, must effectuate steps that if executed correctly will lead to it being absorbed into transcendental phenomenology.” To solve the problem, Husserl—following Zahavi—attempts to hold that the psychologist might adopt the natural attitude *after* the transcendental reduction; by doing so, the psychologist does psychology which is transcendentally founded. Yet, it is hard to define this *new* psychology. Zahavi critically discusses Davidson and Cosgrove's attempt at defining psychology as founded on the “personal attitude,” and Morley's demand that psychology has to employ a methodological metareflection on how to secure the reduction. Zahavi is skeptical about such attempts and he holds that the epoché and the reduction are not essential to non-philosophical applications of phenomenology. At this point, phenomenology stops being a fundament for non-philosophical disciplines, including psychology and cognitive science, but instead it can establish an exchange between various non-philosophical fields. Admittedly, this approach implies a revision of at least some claims of Pokropski, but, again, it seems to be arguably more efficient since it does not require to address the methodological question of how to develop a non-transcendental psychology which uses transcendental tools.

## Author Contributions

WP reviewed the literature, developed the theoretical stance, wrote the manuscript, and prepared to publication.

## Conflict of Interest

The author declares that the research was conducted in the absence of any commercial or financial relationships that could be construed as a potential conflict of interest.
